# COVID-19 with acute myocardial infarction evaluated using multimodal imaging: A case report

**DOI:** 10.1097/MD.0000000000043412

**Published:** 2025-07-18

**Authors:** Jingyao Wang, Yachao Li, Mengjie Lei, Zeyong Guan, Zhigang Zhao, Xiao Wang, Zengming Xue

**Affiliations:** a Department of Cardiology, Langfang People’s Hospital, Hebei Medical University, Langfang Core Laboratory of Precision Treatment of CAD, Langfang, China; b Cardiometabolic Medicine Center, Fuwai Hospital, National Center for Cardiovascular Diseases, Chinese Academy of Medical Sciences and Peking Union Medical College, Beijing, China.

**Keywords:** case report, COVID-19, multimodal imaging, acute myocardial infarction, vasculitis

## Abstract

**Rationale:**

Although the global impact of coronavirus disease 2019 (COVID-19) has diminished, cardiovascular complications, such as acute myocardial infarction, remain a critical concern. This case highlights the potential role of COVID-19 in destabilizing preexisting coronary plaques through systemic inflammation and hypercoagulability, offering insights into diagnostic and therapeutic strategies for similar patients.

**Patient concerns:**

A 41-year-old woman with no prior cardiovascular risk factors presented with sudden-onset chest pain 5 days after a confirmed COVID-19 diagnosis. Initial electrocardiography showed ST-segment changes and troponin I levels were mildly elevated.

**Diagnosis:**

Emergency coronary angiography revealed 90% stenosis in the middle segment of left anterior descending artery with thrombus, confirming acute anterior myocardial infarction. Optical coherence tomography identified vulnerable plaque features, including macrophages and cholesterol crystals, without plaque rupture.

**Interventions and outcomes:**

Thrombus aspiration and optical coherence tomography-guided drug-eluted balloon angioplasty were performed. Post-procedural multimodal imaging (coronary computed tomography angiogram, cardiac magnetic resonance) demonstrated residual plaque burden and mid-myocardial late gadolinium enhancement, suggesting dual ischemic and inflammatory injuries. Intensive lipid-lowering therapy (alirocumab and rosuvastatin) and dual antiplatelet therapy were also administered.

**Lessons:**

The disease course of the patient suggests that COVID-19 could play a role in the destabilization of atherosclerotic plaques through a high inflammatory status in the blood vessels, leading to a hypercoagulable state and acute myocardial infarction induction. Therefore, monitoring inflammatory markers and administering adequate anti-inflammatory treatments may be necessary for such patients.

## 1. Introduction

Although the global infection and mortality rates of the coronavirus disease 2019 (COVID-19) have shown a downward trend, this remains a considerable global health issue. World Health Organization (WHO) data indicate that by April 2025, there were over 777 million cases worldwide, with China ranking second in infection cases.^[1]^ The effect of COVID-19 on the body is not limited to the respiratory tract. It can cause cardiovascular complications in 10% to 30% of cases.^[2]^ Compared to patients with acute myocardial infarction (AMI) without COVID-19, the risk of hospital death in patients with COVID-19 has increased by more than 5 times.^[3]^ The mechanism by which COVID-19 leads to AMI can be summarized as follows: angiotensin-converting enzyme-2 in the myocardium and coronary arteries, leading to local inflammation, hypercoagulability, and thrombosis. Additionally, severe acute respiratory syndrome coronavirus-2 (SARS-CoV-2) directly activates platelets, causing a coagulation cascade that may produce a prothrombotic environment. This leads to a hypercoagulable state, which results in coronary thrombosis and AMI.^[4]^ Myocardial infarction can also be induced by the rupture or erosion of unstable plaques by inflammatory factors, leading to thrombosis.^[5]^ This report describes the case of a young woman who developed AMI after COVID-19. We performed an emergency coronary angiography examination and performed thrombus aspiration and optical coherence tomography (OCT) guided drug-eluted balloon (DEB) angioplasty. After the patient’s condition stabilized, we performed pathological and multimodal imaging examinations to investigate the pathophysiological features of AMI in this COVID-19-associated patient. These observations may provide preliminary insights for future studies of diagnostic and therapeutic approaches in similar clinical scenarios.

## 2. Case description

**Figure 1.**
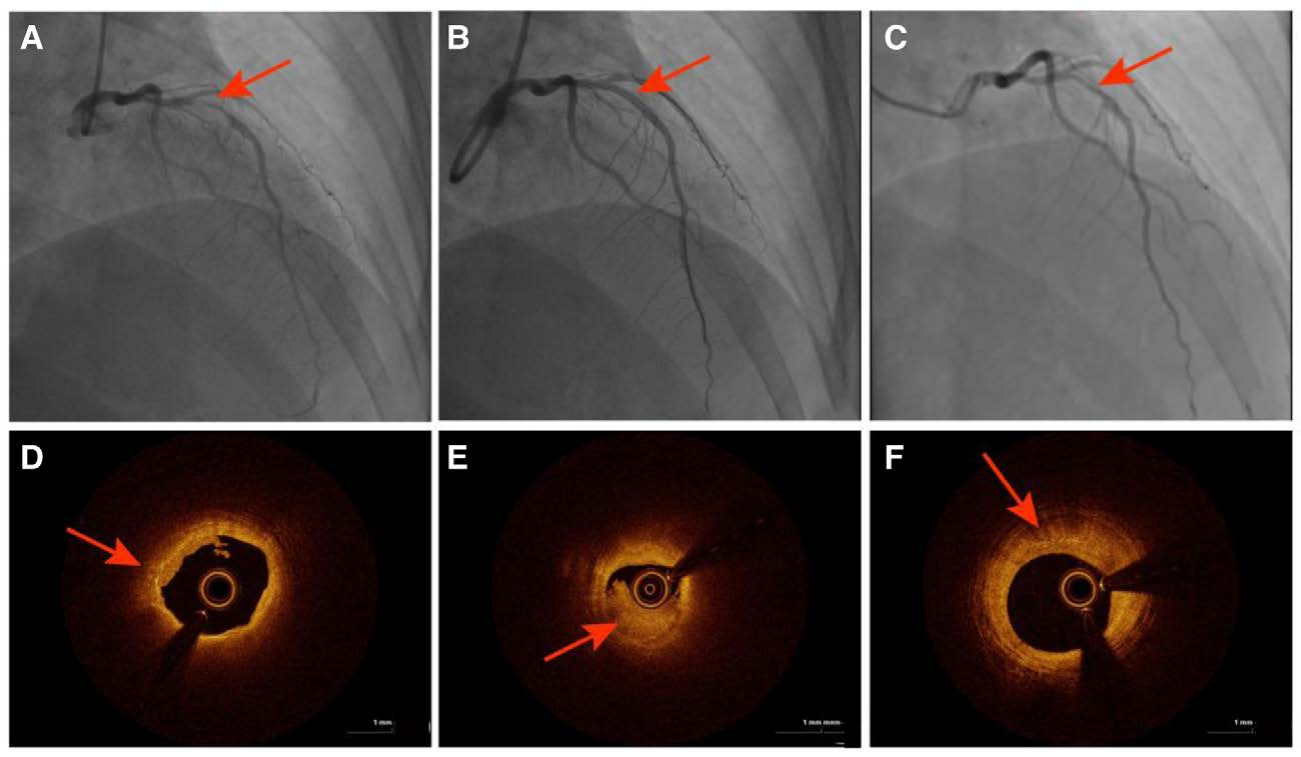
(A and B) The LAD imaging before and after DEB treatment in the patient. (C) The LAD imaging during the 6-month follow-up, and (D–F) cholesterol crystals, thrombus, and macrophages under OCT during the first angiography. DEB = drug- eluted balloon, LAD = left anterior descending, OCT = optical coherence tomography.

**Figure 2.**
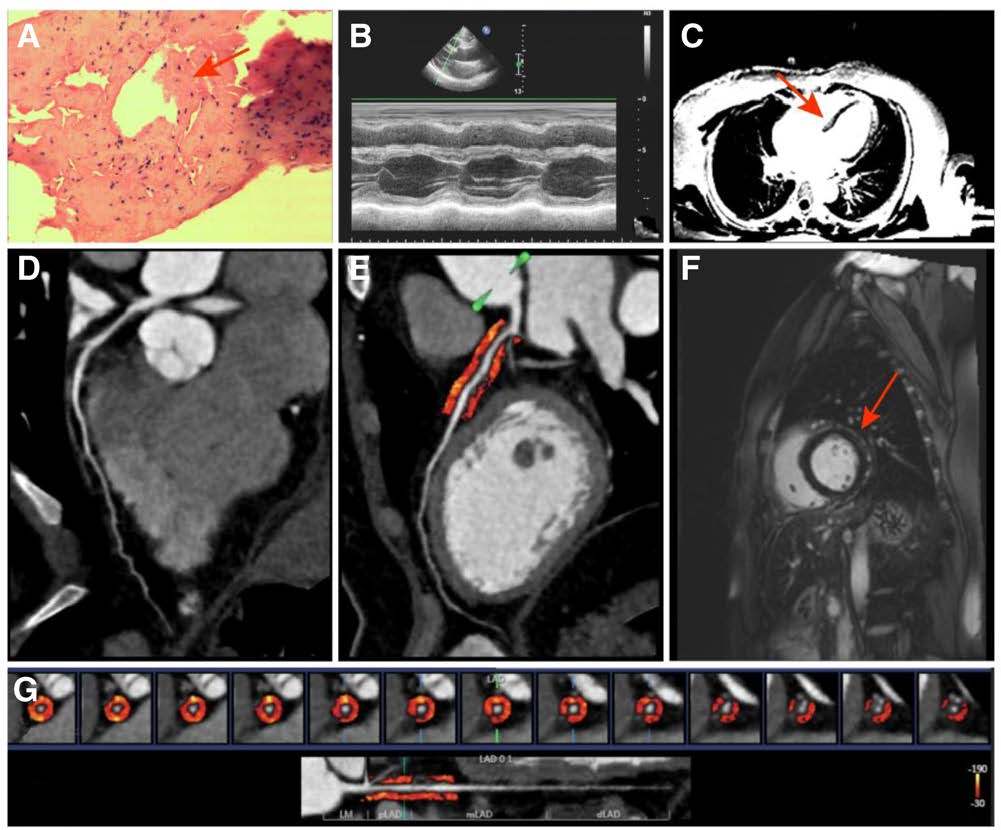
(A) The pathological section of thrombus. (B) The ventricular wall motion in cardiac ultrasound. (C and F) The myocardial magnetic resonance imaging results. (D, E, and G) The CTA and FAI results of the patient’s LAD. CTA = computed tomography angiogram, dLAD = distal LAD, FAI = fat attenuation index, LAD = left anterior descending, LM = left main lesion, mLAD = middle LAD, pLAD = proximal LAD.

A 41-year-old woman was admitted to our hospital at 11: 31 am on March 11, 2024, because of persistent chest pain for 4 hours. The patient had no history of coronary heart disease, hypertension, or diabetes mellitus. The patient had no family history of coronary heart disease. She had no history of smoking or alcohol consumption. Five days earlier, she had been diagnosed with COVID-19 at a community hospital. Four hours prior to admission, the patient suddenly experienced chest pain while cooking. The patient visited the emergency department because of persistent symptoms. Electrocardiography revealed sinus rhythm with a heart rate of 64 beats per minute, ST-segment depression of 0.1 mV in leads I, II, III, aVL, and V4 to V6, and ST-segment elevation of 0.1 mV in lead aVR. No increase in high-sensitivity troponin I level was observed. Immediate emergency coronary angiography revealed 90% stenosis in the middle segment of the left anterior descending (LAD) artery, with a visible thrombus shadow and an initial thrombolysis in myocardial infarction (TIMI) flow grade III (Fig. 1A). Therefore, thrombus aspiration was performed in the LAD artery and a large white thrombus was extracted for pathological examination. Post-thrombus aspiration and TIMI flow remained grade III, and OCT (Abbott Laboratories, USA) examination revealed fibrous lipid plaques, visible thrombus (mainly white thrombus), macrophages, and cholesterol crystals at the lesion site of the LAD artery. No significant plaque rupture was observed, with a minimum lumen area of 1.02 mm^2^ and a relative stenosis rate of approximately 70% (Fig. 1D–F). After pretreatment at the lesion site, 1 RESTOREDEB 2.5 × 25 mm (Cardionovum GmbH, CHN) was applied. Post-procedure OCT examination revealed no significant dissection or hematoma at the lesion site, with visible macrophages, cholesterol crystals, and drug delivery in the intima and a minimum lumen area of 3.52 mm^2^ after DEB angioplasty. Post-procedure TIMI flow grade III was achieved (Fig. 1B). The patient was treated with aspirin and ticagrelor as antiplatelet therapy, as well as alirocumab and rosuvastatin calcium as lipid-lowering therapies. Post-procedure laboratory tests showed a monocyte count of 0.76 × 10^9^/L, low-density lipoprotein (LDL) 2.96 mmol/L, positive novel coronavirus nucleic acid test (N gene cycle threshold [Ct] value: 30.37; ORF1ab gene Ct value: 29.15), and troponin I 0.089 μg/L (reference value 0.010–0.023 μg/L). Pathological examination of the aspirated thrombus revealed transparent, structureless, red-stained fibrin with scattered cells and focal calcium salt deposition, consistent with thrombosis (Fig. 2A). An echocardiogram was performed on the first post-procedure day, which demonstrated only mild mitral valve regurgitation without other significant abnormalities. Coronary computed tomography angiogram performed 7 days after procedure indicated no calcification in both coronary arteries, right-sided dominant coronary artery, and mild stenosis in the proximal lumen of the anterior descending branch (Fig. 2D). CT fractional flow reserve values were 0.91 (LAD), 0.94 [left circumflex artery (LCX)], and 0.96 [right coronary artery (RCA)]. The pericoronary fat attenuation index (FAI) values were −77 (LAD), −79 (LCX), and −85 (RCA) (Fig. 2E and G). Echocardiography performed 7 days after procedure revealed normal atrial and ventricular dimensions, normal wall thickness, and motion amplitude, and minor mitral and tricuspid valve regurgitation (Fig. 2B). Cardiac magnetic resonance imaging 9 days after procedure indicated late gadolinium enhancement (LGE) in the mid-myocardial layer of the basal anterior, basal septal, basal inferior, and mid-anterior segments, as well as at the right ventricular inferior insertion point (Fig. 2C and F). The patient’s condition improved after treatment, and she was discharged. The patient did not experience chest pain during follow-up, and no novel coronavirus reinfection occurred. On August 14, 2024, the patient was admitted for follow-up coronary angiography with an LDL level of 1.39 mmol/L. Coronary angiography revealed a plaque in the proximal segment of the anterior descending branch, 70% stenosis in the middle segment of the lumen, and no significant stenosis in the RCA or the circumflex branch. Follow-up angiography (Fig. 1C) showed TIMI flow grade III in all vessels, and because the patient refused to undergo intraprocedural endovascular imaging and functional studies, a cardiopulmonary exercise test was performed after angiography and revealed a negative electrocardiogram stress test before discharge. Follow-up to date has shown no recurrence of the chest pain.

The specific timeline of onset is shown in Table 1.

**Table 1:** Timeline.

Time line	Intervention measures
March 11, 2024 11:31 am	Be hospitalized.
March 11, 2024 11:50 am	PerformedDEB dilatation under optical coherence tomography guidance.
March 14, 2024	Post-procedure pathological report.
March 18, 2024	Coronary CTA was performed post-procedure.
March 18, 2024	Echocardiography after surgery.
March 20, 2024	Post-procedure cardiac magnetic resonance imaging.
August 15, 2024	Coronary angiography re-check.

CTA = computed tomography angiogram, DEB = drug-eluted balloon.

## 3. Discussion

This case involved a young woman with stable coronary artery disease (evidenced by preexisting fibrous lipid plaques on OCT) who developed acute coronary syndrome following COVID-19. The rapid progression of symptoms was likely triggered by the inflammatory cascade induced by SARS-CoV-2 infection, which destabilizes preexisting atherosclerotic plaques. Notably, OCT revealed macrophages and cholesterol crystals at the lesion site, which are both markers of plaque vulnerability. Perivascular FAI values (−77 to −85 HU) further indicated a systemic inflammatory state. These findings suggest that COVID-19 exacerbates vascular inflammation and promotes plaque erosion and thrombosis, rather than directly causing de novo coronary stenosis.

### 3.1. Chronic inflammatory substrate of coronary disease

The patient’s preexisting coronary stenosis was fundamentally attributable to chronic vascular inflammation, as evidenced by OCT findings, FAI values, and LDL levels. OCT imaging revealed macrophages and cholesterol crystals at the LAD lesion site, both of which exacerbated atherosclerosis through inflammatory pathways. Macrophages promote early atherosclerotic lesions via foam cell formation, which is driven by inflammation and lipid phagocytosis.^[6]^ Cholesterol crystals activate the *NOD-like* receptor family pyrin domain containing 3 inflammasome, triggering interleukin-1β production, and their accumulation within plaques compromises stability through associated inflammation.^[7]^

Elevated attenuation of FAI further indicates systemic inflammation. Studies have confirmed that coronary arteries with high inflammatory activity release signals to the perivascular adipose tissue, inhibiting local adipogenesis and resulting in high FAI attenuation.^[8]^ Cross-sectional data demonstrate significantly higher FAI in AMI patients (-82.3 ± 5.5 HU) versus non-coronary artery disease controls (-95.8 ± 6.2 HU, *P* < .001) and stable coronary artery disease patients (-90.6 ± 5.7 HU, *P* < .001).^[9]^ This patient’s pan-coronary inflammation was reflected in FAI values of −77 HU (LAD), −79 HU (LCX), and −85 HU (RCA).

Additionally, an admission LDL level of 2.96 mmol/L classified her as high-risk for atherosclerotic cardiovascular disease. Intensive lipid-lowering therapy (alirocumab + rosuvastatin) reduced LDL to 1.39 mmol/L at 6-month follow-up. However, the rapid progression of the LAD lesion underscores that LDL reduction alone is insufficient to suppress vascular inflammation, with OCT/FAI features indicating persistent inflammatory activity.

### 3.2. COVID-19 as the acute precipitant of AMI

Acute inflammation due to COVID-19 likely triggered this AMI event. Retrospective studies have shown that COVID-19-associated AMI involves a higher thrombus burden (increased thrombus aspiration rates and stent thrombosis),^[10]^ consistent with our angiographic findings. SARS-CoV-2 promotes endothelial injury through angiotensin-converting enzyme-2 receptor binding, causing receptor downregulation, elevating angiotensin II activity, and inducing vasoconstriction, apoptosis, inflammation, hypercoagulability,^[4]^ and endotheliitis, which disrupts vascular homeostasis.^[11]^

Plaque vulnerability is exacerbated by the direct viral infection of intraplaque macrophages/foam cells, which amplifies local inflammation.^[12]^ Pathological analysis of the aspirated thrombus (fibrin with calcium deposition, no inflammatory cells) aligns with acute thrombosis secondary to plaque erosion. The core pathophysiology of plaque erosion involves inflammatory cytokine activation (e.g., minimally oxidized LDL and hyaluronic acid fragments), which upregulates interstitial collagenases (e.g., MMP-2), degrading the basement membrane and causing endothelial dysfunction/desquamation.^[13]^ SARS-CoV-2 infection likely triggers this inflammatory cascade.

### 3.3. Dual mechanism of myocardial injury

Postoperative cardiac magnetic resonance suggested combined ischemic and inflammatory injury: LAD-territory LGE indicated ischemic damage, and mid-myocardial LGE in non-coronary distributions (basal anterior/septal/inferior walls) suggested concomitant myocarditis. COVID-19 can directly cause myocardial injury via inflammatory myocarditis, characterized by variable endocardial/epicardial LGE, elevated T2, and STIR positivity.^[14]^ In this case, LGE sparing of the endocardium may reflect: (1) nonocclusive coronary disease causing limited ischemic injury; (2) the potential COVID-19-related myocardial inflammation contributes to troponin elevation, and myocardial biopsy is required to confirm the inflammatory etiology.

Although COVID-19 acted as an immediate trigger, rapid plaque progression in this young patient raises suspicion for undiagnosed systemic disorders (e.g., autoimmune vasculitis or coagulopathy). We recommended screening for antinuclear antibodies, antiphospholipid antibodies, and lipoprotein (a) levels; however, the patient declined further testing during follow-up owing to personal scheduling conflicts.

## Author contributions

**Conceptualization:** Jingyao Wang, Mengjie Lei, Zengming Xue.

**Formal analysis:** Yachao Li, Zeyong Guan, Zhigang Zhao, Zengming Xue.

**Funding acquisition:** Yachao Li, Zhigang Zhao, Zengming Xue.

**Investigation:** Yachao Li, Zeyong Guan, Zhigang Zhao, Xiao Wang, Zengming Xue.

**Software:** Jingyao Wang, Mengjie Lei, Zeyong Guan, Xiao Wang.

**Supervision:** Jingyao Wang, Yachao Li, Mengjie Lei.

**Validation:** Mengjie Lei.

**Writing – original draft:** Jingyao Wang.

**Writing – review & editing:** Jingyao Wang.
